# Complement Inhibition in Chronic Subdural Hematoma Fluid

**DOI:** 10.1007/s10753-024-02210-3

**Published:** 2024-12-09

**Authors:** Niklas Marklund, Shaian Zolfaghari, Gustaf Westerberg, Karsten Ruscher, Elisabet Englund, Henrietta Nittby Redebrandt

**Affiliations:** 1 Department of Clinical Sciences Lund, Division of Neurosurgery, Department of Neurosurgery, Lund University and Skane University Hospital, Lund, Sweden; 2 Department of Clinical Science, Division of Pathology, Lund University, Lund, Sweden

**Keywords:** Chronic Subdural Hematoma, Biomarker, Complement Factor

## Abstract

**Background:**

Emerging data suggest a complex pathophysiology of chronic subdural hematoma (CSDH) to which an inflammatory response might contribute. The complement system is activated in acute traumatic setting, although its role in CSDH is unknown. To investigate the complement system in CSDH pathophysiology, we analyzed blood and hematoma fluid biomarkers, as well as immunohistochemistry of the CSDH membrane and dura.

**Materials and Methods:**

We simultaneously collected CSDH fluid and peripheral blood from 20 CSDH patients at the time of surgery. Biopsies of the dura mater and the CSDH capsule were obtained and analyzed by immunohistochemistry for C5b-C9 or C5a deposition. Biomarkers of inflammation and complement activation were analyzed by a 21-multiplex assay, including Adiponectin, Clusterin, Complement factor C9 and CRP. Complement factor C5a was analyzed separately by a commercial R-plex electrochemiluminescence assay.

**Results:**

Ten biomarkers differed significantly between peripheral blood and paired CSDH of which two were significantly increased in CSDH fluid (Clusterin and Cystatin C). Eight of the significantly altered biomarkers were significantly decreased in CSDH fluid, including C5a, Complement 9 and Adiponectin. There was no immunoreactivity for C5a or the C5b-C9 membrane attack complex in the dura or CSDH membrane.

**Conclusions:**

In CSDH levels of the complement inhibitor Clusterin were increased, whereas levels of C5a and C9 were decreased. Membrane attack complex C5b-C9 was not detected in the membrane or dura surrounding the CSDH. Inhibition of complement could lead to reduced clearance of debris in the CSDH as well as secondary inflammatory reactions.

## Introduction

The incidence of chronic subdural hematoma (CSDH) has increased markedly in recent years, both of operated and non-operated cases [1]. In symptomatic patients, surgical evacuation is the gold standard, with neuro-interventional therapies gaining increased relevance [2, 3]. Patients treated for CSDH have excess mortality that correlates with poor modified Rankin score upon admission and discharge, alcohol abuse and age ≥ 80 years, among other factors [4].

The surgical treatment of CSDH is not without risk, and serious complications occur in a subset of patients. Moreover, the recurrence rate ranges from 9–33% [5–7], with risk factors including bilateral CSDH and pre-operative width of the hematoma [5]. While the use of body-tempered irrigation fluid reduced the risk of re-operation [8], there is no effective and safe stand-alone medical treatment against CSDH. Dexamethasone reduced the recurrence rate, but was associated with more complications than in those treated with surgery alone, as well as an increased risk of later surgery [9].

The incidence of patients presenting with CSDH will likely continue to increase due to an ageing population as well as the increasing use of anti-thrombotic drugs [5]. Only 0.13% of patients with an initially normal CT scan following a head injury later developed a CSDH that required surgery [10]. There are several hypothesis regarding the formation of CSDH, including a state of chronic inflammation and excessive fibrinolysis [11]. It is since long established that inflammatory cytokines such as interleukin (IL)−6 (IL-6) and IL-8 are increased in the CSDH fluid when compared to peripheral blood [11, 12]. A recent analysis demonstrated an increase both of pro-inflammatory dendritic cells and anti-inflammatory macrophages in the CSDH compared to peripheral blood [13].

In traumatic brain injury (TBI) the function of the coagulation system is impaired, leading both to increased bleeding in some situations, but also hyper-coagulation [14]. It is well known that coagulation is linked to the complement system and cross-talk between these systems can lead to both activation and inhibition [15]. The complement system consists of more than 30 proteins and several regulators that are activated upon initiation of a complement-mediated response [16]. The complement systems function as a bridge between innate and adaptive immune response, and helps eliminating pathogens in blood and tissue. The complement system has three pathways, the classical, the alternate and the lectin mediated pathways [16]. These pathways converge at the level of C3 cleavage, where C3 convertase cleaves C3 into C3a and C3b. C3a leads to inflammation, whereas C3b leads to opsonization of pathogens. Furthermore, the cascade leads o cleavage of C5 into C5a and C5b, where C5a is an anaphylatoxin and C5b binds to C6-C7-C8-C9 to form the membrane attack complex (MAC) [16].

The aim of the present study was to define proteins that differ in CSDH fluid compared to peripheral blood from the same patients upon neurosurgical intervention, with focus on complement associated biomarkers. In addition, dura and hematoma capsule were analyzed for C5a and C5b-C9 deposition.

## Materials and Methods

### Ethics Statement

The study was approved by the Swedish Ethics Review Authority (permit number 2020—03154). Patients were included after signed informed consent in accordance to the ethical permission. The study was performed in accordance with the Declaration of Helsinki.

### Patients and Surgical Procedure

Symptomatic patients who were admitted to the Department of Neurosurgery in Lund, Skåne University Hospital, Sweden in order to undergo evacuation of a CSDH in 2022, were asked for informed consent to participate in the study. Patients were excluded if they had an ASDH or if peripheral blood and CSDH fluid could not be obtained simultaneously. All included patients had a CT-verified CSDH requiring surgical evacuation. The CSDHs were evacuated in local anesthesia under light sedation with Remifentanil, which was initiated once the surgical procedure started. One to three burr holes were made in the skull and the dura was opened as previously described by us [7]. Dura and CSDH membrane samples were collected when possible, but in some cases it was not possible for technical reasons e.g. due to dural bleeding or when the burr hole was too small for proper sampling possibility. After collection of CSDH fluid for analysis (vide infra), irrigation by room tempered sodium chloride was performed until no visible hematoma fluid was remaining. A subgaleal drain was placed and kept in place for 24 h post-operatively according to the standard routine adopted by our department. Patients were monitored for at least 24 h after the surgery. No routine CT control was ordered unless there was a clinical indication, in accordance to our standard clinical practice.

### Collection of Blood, Hematoma Fluid and Tissue Biopsies

Immediately prior to the initiation of the surgical procedure, in the operating theatre prior to injection with the local anesthetic and the surgical incision, a 10 cc peripheral venous serum sample was collected. After opening of the dura, 10 cc of CSDH hematoma fluid was collected in a sterile sampling tube for later analysis. After fluid sampling and tissue biopsy (vide infra), the procedure continued according to standard clinical practice. The hematoma samples were immediately stored at 4 °C after sampling, after which they were centrifuged as soon as the surgery was finished.

Peripheral blood and CSDH fluid were centrifuged at 4000 rpm for 10 min at 4 °C. The supernatant was collected and stored in a −80 °C freezer until analysis. All samples were analyzed at the same occasion.

### Multiplex Analysis

A U-PLEX Biomarker Group 3 Human 21-Plex (MESO SCALE DIAGNOSTICS, LLC. Rockville, MD USA) was used for analyses. The assay contained three plates analyzing the following biomarkers: a 4-Plex assay was used to measure the levels of SHBG, sTfR-1, VCAM-1 and vWF, respectively, the 7-Plex assay covered A2M, Adiponectin, ApoA1, ApoC3, Complement C9, RBP4 and Serpin A1, the 10-Plex assays included CA1, Clusterin, Complement C9, Complement factor D, CRP, Cystatin C, DPPIV, Factor VII, ICAM-1, NGAL/LCN2, RBP4, SAA, respectively.

Briefly, samples were thawed on ice and further equilibrated to room temperature. Hematoma samples were centrifuged at 20800xg at 4˚C for 10 min. Samples used to analyze CA1, clusterin, Complement Factor D, CRP, Cystatin C, DPPIV, Factor VII, ICAM-1, NGAL/LCN2, SAA, SHBG, STfR-1, VCAM-1 and vWF were diluted 1:4000 prior to use.

Subsequently, all steps of the assays were performed according to the manufacturer’s instructions. Following coating in respective solutions at room temperature for 1 h, plates were washed with 150 µl/well 1 × Wash Buffer (phosphate buffered saline containing 0.1% Tween 20). Thereafter, samples or calibrators/standards (50 µl/well) were added, plates were sealed, and incubated at room temperature with shaking (1000 rpm) for 2 h. Plates were washed three times. Thereafter, plate specific detection antibody solutions (50 µl/well) were added, plates sealed and incubated at room temperature with shaking for additional 2 h. Plates were again washed three times in 1 × Wash buffer. Prior to loading the plate for analysis, a 2 × MSD Gold Read Buffer T (150 µl/well) was added to the plate. Reading was accomplished on a MESO QuickPlex SQ 120 instrument and analysis performed using the MSD Discovery Workbench software version 4.0.13 (Rockville, MD, USA). All samples were analyzed in duplicates, on the same batch and by the same researcher experienced with the technique (KR).

A single R-plex assay was used to analyze C5a (Mesocale, Rockville, USA). Plates were coated with biotinylated capture antibody for one hour at room temperature. Washing of plates were accomplished in between every of the subsequent steps with 1 × wash buffer three times 150µL/each well). Thereafter, plates were loaded with 25 µL assay diluent and immediately after, either with 25 µL of sample or calibrator and incubated at room temperature for one hour. Fifty microliters of detection antibody solution were added to each well and plates were incubated at room temperature for one hour. Finally, 2 × MSD Gold Read Buffer T (150 µl/well) was added to the plate and reading was performed on a MESO QuickPlex SQ 120 instrument, analysis on the MSD Discovery Workbench software version 4.0.13 (Rockville, MD, USA). All samples were analyzed in duplicates on the same occasion using the same batch.

### Histology and Tissue Analysis

Dural samples and CSDH membrane were collected at time of CSDH surgery. The samples were stored in paraformaldehyde and sent to Department of Pathology, Skåne University Hospital, Lund, for further preparations. Samples were paraffin embedded, sectioned and mounted. Deparaffinization was performed followed by antigen retrieval. Staining was performed with anti-C5a-antibody (anti-human Complement C5a antibody, Thermo Fisher, USA) (30 µg/ml) or anti-C5bC9-antibody (anti-human C5b-C9 antibody, Thermo Fisher, USA) (1:400 concentration), and using the ABC-technique with Vectastain secondary antibody (Vector Laboratories, USA) with DAB as a chromogen. Counterstaining was done with hematoxylin. Immunostained samples were visualized on an Olympus VS-120-S6 virtual slide microscope under a 20X objective according to the manufacturer’s instructions.

### Statistics

SPSS was used for all statistical analyses (IBM SPSS Statistics version 27.0, New York, USA). Correlations were tested with Pearson test. The results of the biomarker assays were tested for normality with Shapiro–Wilk’s test. The exact Wilcoxon signed rank test was used to compare biomarkers between CSDH fluid and matched peripheral blood samples from the same patients and a post-hoc Bonferroni correction was applied to adjust for multiple hypothesis testing (2-sided test, p < 0.05 for statistical significance). Images were generated in Prism (Boston, USA).

## Results

### Patient Characteristics

We included 20 recruited patients (Table 1), with a mean age of 73 years (SD 10 years). Two patients (10%) had previous neurosurgical treatment within a period of three months preceding study inclusion – one patient had been operated due for a CSDH and the other patient had been operated due to an acute subdural hematoma (ASDH). All other patients underwent their primary CSDH surgery (90%). All patients were fully awake and had a Glasgow Coma Scale (GCS) score of 14–15. Four patients had bilateral CSDHs, two of whom underwent bilateral evacuation. In the case of bilaterally evacuated CSDHs, the sample was collected from the first hematoma that was evacuated. All patients were operated with one to three burr holes, according to our standard clinical practice. Two patients (10%) were subsequently re-operated due to a recurring CSDH within three months. One patient had a post-operative superficial wound infection within three months requiring antibiotics but no surgical intervention. One patient suffered from post-operative neurological impairment due to epileptic seizures within 48 h after surgery.

**Table 1:** Patient characteristics

Patient characteristics	
Age (mean ± SD)	73 ± 10
Sex (female n (%))	4 (20%)
Usage of anti-thrombotics pre-operatively	9 (45%)
Pre-operative Markwalder score 1	6 (30%)
Pre-operative Markwalder score 2	13 (65%)
Paresis pre-operatively (n)	10
Maximal thickness of hematoma (mm) (mean ± SD)	21 ± 1
Midline shift (mm) (mean ± SD)	7 ± 1

### Biomarkers

The 21 U-plex biomarkers were analyzed using CSDH fluid from 20 patients and peripheral blood from the same 20 patients. In addition, R-plex assay was run for C5a on hematoma from 19 patients and peripheral blood from 19 patients (there is missing data from one hematoma sample due to a technical error at time of analysis). Serpin A1, ApoA1 and CA1 statistics could not be performed, due to few samples being above the level of detection (< three valid data sets in either CSDH or peripheral blood), leaving 19 biomarkers for analysis. The remaining variables were analyzed in terms of distribution and data was not normally distributed.

**Fig. 1:**
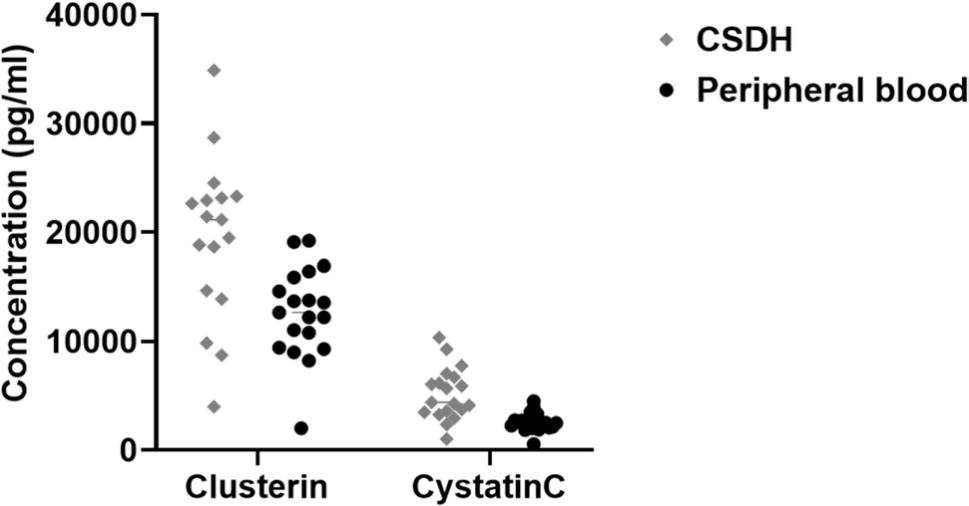
Increased levels of proteins in CSDH when compared to peripheral blood

**Fig. 2:**
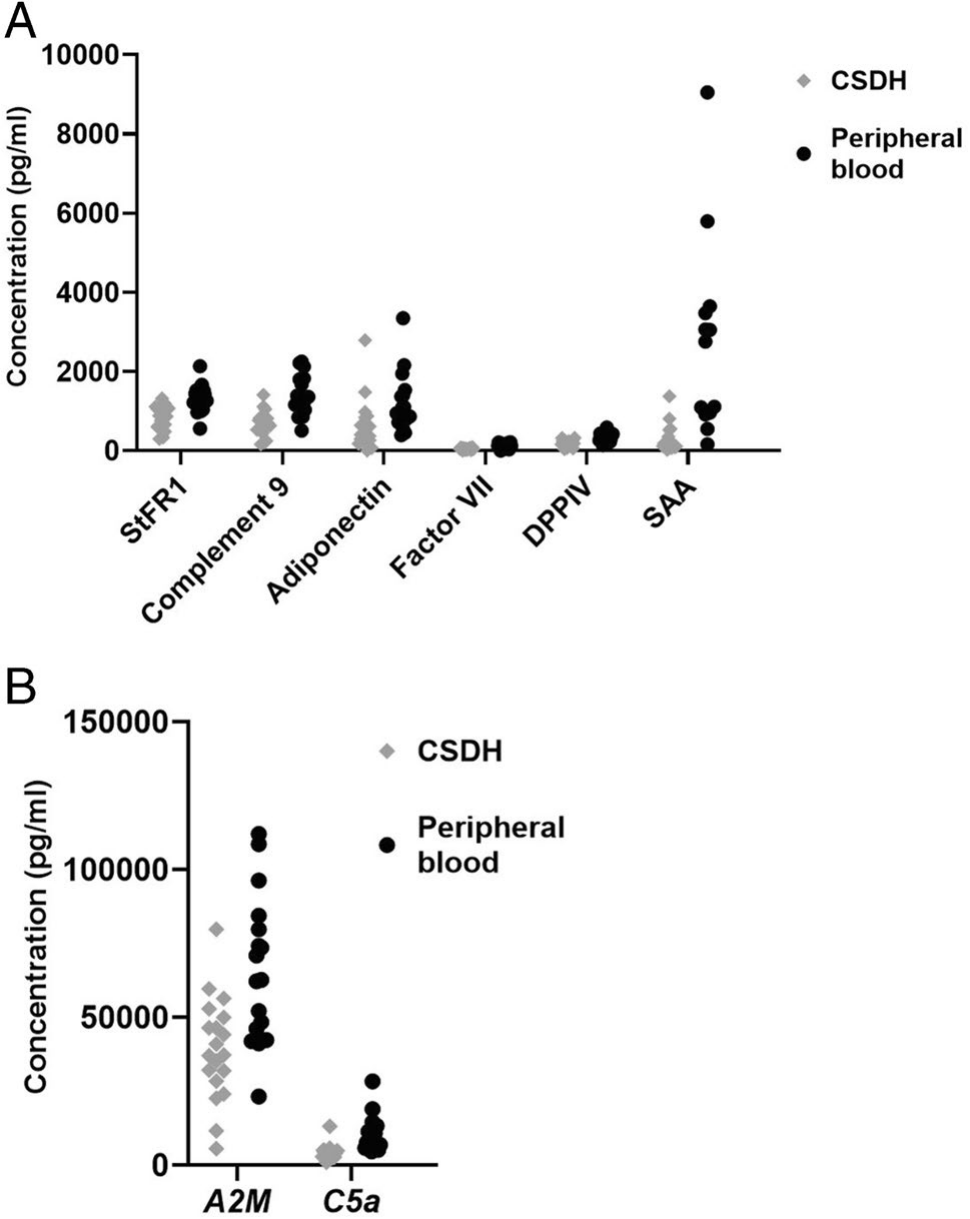
**a** Decreased biomarkers in CSDH compared to peripheral blood. **b.** Decreased biomarkers in CSDH compared to peripheral blood

Ten biomarkers in the multiplex assay differed significantly between peripheral blood and CSDH fluid, collected from the same patients at the same time. Two biomarkers were significantly increased in the CSDH fluid (Clusterin and Cystatin C) (Table 2, Fig. 1), nine were not significantly altered (Table 3) and eight biomarkers were significantly decreased in CSDH compared to levels in peripheral blood (StFR1, Complement 9, A2M, Adiponectin, Factor VII, DPPIV, SAA, C5a) (Table 4, Fig. 2).

**Table 2:** Individual protein levels that were significantly increased in CSDH fluid when compared to peripheral blood

Biomarkers levels significantly increased in CSDH compared to peripheral blood	Bonferroni-adjusted *p*-value (2-tailed)
Clusterin	0.032*
Cystatin C	0.002*

**Table 3:** Proteins that were not significantly altered in CSDH fluid when compared to peripheral blood

Biomarkers not significantly altered in CSDH compared to peripheral blood	Bonferroni adjusted p-value (2-tailed)
SHB	1.0
VCAM1	1.0
vWf	1.0
RBP4	0.60
ApoC3	0.76
Complement factor D	1.0
ICAM1	1.0
NGA/LCN2	0.40
CRP	1.0

**Table 4:** Protein levels that were significantly decreased in CSDH compared to peripheral blood

Biomarkers significantly decreased in CSDH compared to peripheral blood	Bonferroni-corrected *p*-value (2-tailed)
StFR1	0.0003*
Complement 9	0.006*
A2M	0.006*
Adiponectin	0.01*
Factor VII	0.001*
DPPIV	0.005*
SAA	0.009*
C5a	0.008*

There was no statistically significant correlation between increased levels of Clusterin or Cystatin C in CSDH fluid when compared to levels in peripheral blood (Pearson correlation p > 0.05).

There was no statistically significant difference in the biomarker levels, neither in CSDH fluid nor in peripheral blood, comparing patients who had undergone previous SDH surgery to those who had not.

These biomarkers are presented separately due their higher levels compared to the other biomarkers.

### Complement Associated Factors

**Fig. 3:**
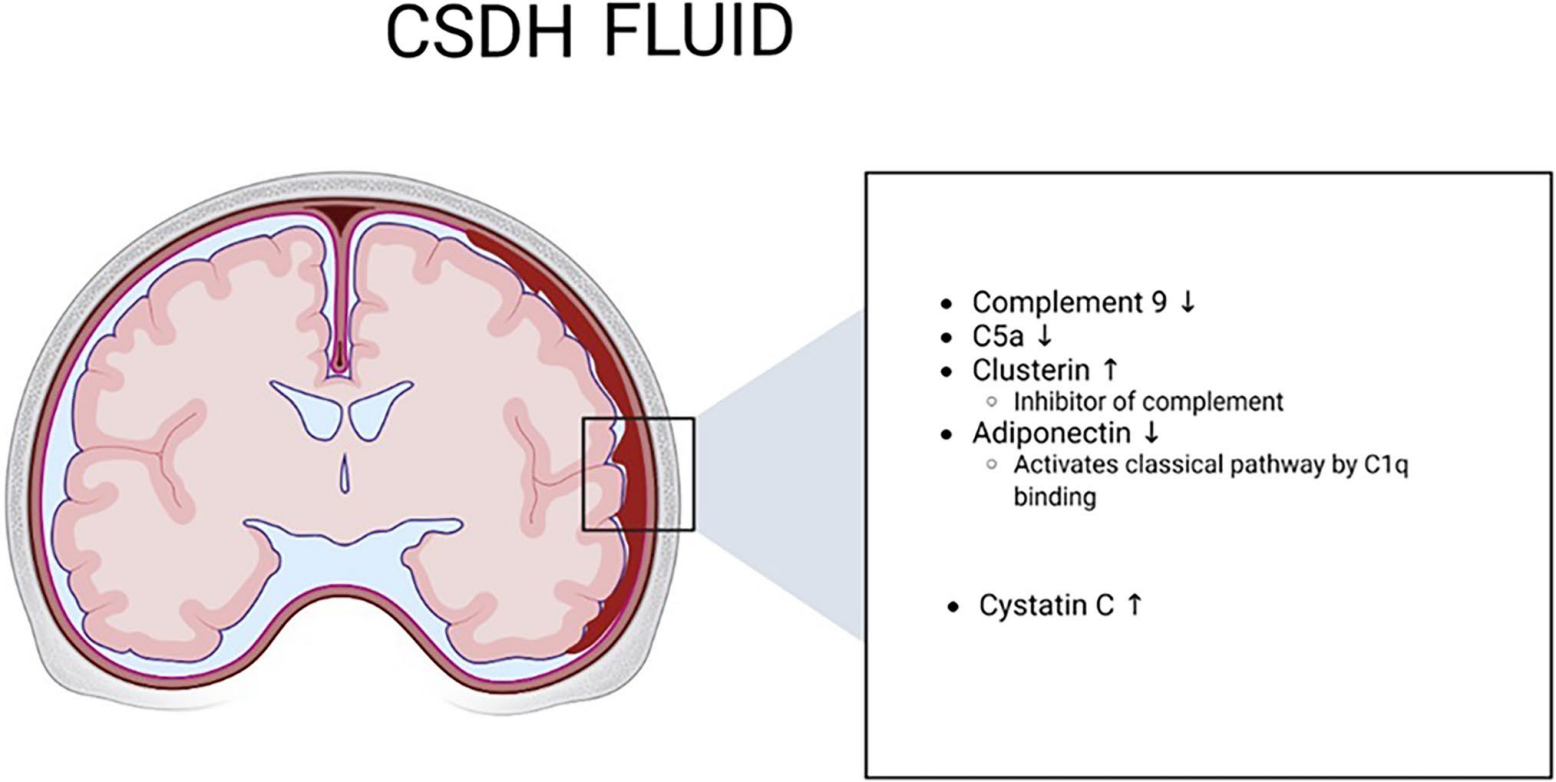
Proteins in the CSDH fluid in relation to peripheral blood sampling. Image created with Biorender (Toronto,Canada). ↓ decreased levels in CSDH fluid when compared to peripheral blood. ↑ increased levels in CSDH fluid when compared to peripheral blood

As presented above, results of complement proteins indicated that there might be a stronger complement inhibition in CSDH compared to peripheral circulation, with low levels of C9 and C5a, whereas the complement inhibitor Clusterin was increased, and complement activator such as Adiponectin was decreased. A summary of complement associated alterations in CSDH is presented in Fig. 3.

### Immunohistochemistry

**Fig. 4:**
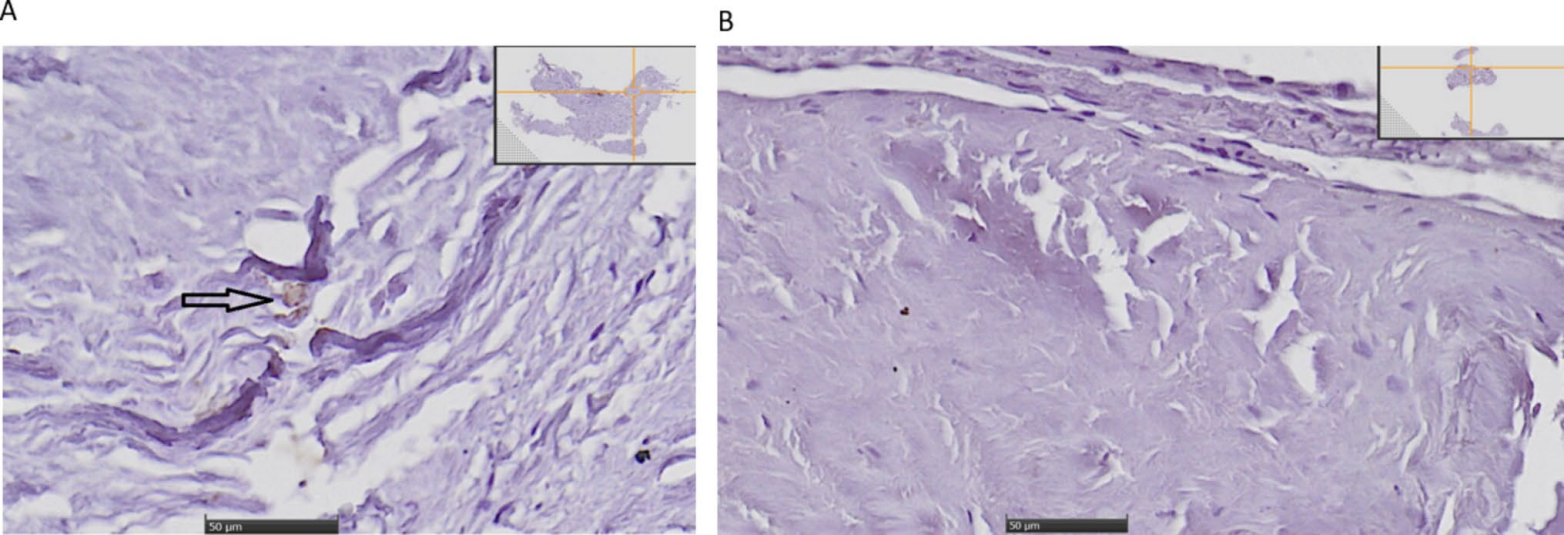
**a** Anti-C5a staining of dura and adjacent capsule. Only occasional immune-reactivity against C5a could be detected (arrow), whereas most of the sample was negative for anti-C5a-antibodies. Scale bar 50 µm. Scout image in right upper corner. **b** Anti-C5b-C9 staining of dura. No detection of C5b-C9 could be observed. Scale bar 50 µm. Scout image in right upper corner

Dura and membrane samples were collected and stained with anti-C5a antibody (Fig. 4a). Only punctate and irregular staining with C5a could be demonstrated, whereas most of the tissue was negative for anti-C5a staining.

There was no immunoreactivity for C5b-C9 in any of the analyzed tissue (Fig. 4b).

In negative controls no primary antibody was added, but secondary antibody and DAB staining was applied. No DAB staining could be demonstrated, as expected (data not shown).

## Discussion

In this study, we compared fluid biomarkers from CSDH to the levels in peripheral blood from the same patients. Our main findings were that two biomarkers were significantly increased in the CSDH fluid compared to peripheral blood (Clusterin and Cystatin C), which may indicate local production or accumulation of these factors. Eight biomarkers were significantly decreased in the CSDH fluid compared to peripheral blood (StFR1, Complement 9, A2M, Adiponectin, Factor VII, DPPIV, SAA and C5a). In summary, the complement inhibitor Clusterin was increased in CSDH fluid, whereas the complement factors C9 and C5a were decreased in CSDH fluid. C5b-C9 was not detected on the dura and membrane. Together, this could indicate lack of complement activity in the CSDH compartment upon the time point of surgery.

Previous studies of complement factors in CSDH fluid are sparse. In one study, protein content in the hematoma from five patients with CSDH was compared to serum using electrophoresis and mass spectrometry, demonstrating a decreased concentration of complement components C4a/b [17]. Complement 9 is a part of the final step of the complement cascade, participating in the formation of the membrane attack complex (MAC) [18]. Activation of complement leads to a proteolytic cascade, with cleavage of C5 into C5a and C5b [19]. C5b is involved in the formation of MAC [19]. The MAC is formed in a sequential pathway including the following steps: C5b → C5b-6 → C5b-7 → C5b-8 and C5b-9 [18]. In situations of complement deficiency, impaired clearance of apoptotic cells and secondary necrosis may activate dendritic cells and a pro-inflammatory response [20]. C5a, generated upon C5 cleavage, is an anaphylatoxin released at sites of inflammation [21]. Adding C5a to human umbilical cord epitheloid cells that express C5a Receptor (C5aR) lead to upregulation of the inflammatory mediators IL-8 and IL1beta, and downregulation of IL-6 [21]. In the present study, C5a was reduced in the CSDH fluid compared to peripheral blood and moreover only scarce C5a immunoreactivity could be detected in the dura and membrane.

In the present study, Clusterin was significantly increased in CSDH fluid compared to peripheral blood. Clusterin is a regulator of the complement system, as it binds to the complexes C5b-C6-C7, or C5b-C6-C7-C8 or C5b-C6-C7-C8-C9 [22]. Upon binding to these complexes, Clusterin renders them lytically inactive [22]. Adiponectin, which can function as a complement activator, was on the other hand decreased in the CSDH fluid in our material [23].

Cystatin C, a Cathepsin B inhibitor [24], was significantly increased in the CSDH fluid in this study. Cathepsin B, in turn, is released after TBI and contributes to neuronal death. Cystatin C is synthesized by the choroid plexus in CNS, and concentrations of Cystatin C in CSF have been reported to be five-fold higher than in peripheral blood [25]. Potentially, the increased level of Cystatin C in the CSDH fluid could be due to CSF contributing to the CSDH.

A2M was significantly decreased in CSDH fluid in our study. A2M is increased during early phases of inflammation and can capture and prevent active proteases from functioning [26]. A2M interacts with proteins of the complement system, for example the lectin pathway of the complement system, although the exact mechanism is not fully known [26]. A2M also increases the phagocytic and anti-microbial capacity of macrophages [26].

A recent study demonstrated that all cells that were identified in peripheral blood in patients with CSDH could be identified in CSDH fluid as well, but at different concentrations [27]. There was no difference in the concentration of platelets, granulocytes or lymphocytes in patients that later developed a recurring CSDH compared to those who did not need a re-operation, whereas inflammatory cells differed between primary surgery and re-operation [27]. Another study compared peripheral blood from CSDH patients to control individuals, demonstrating that all inflammatory indexes were within normal range in both groups [28]. Still, white blood cell count and neutrophil counts were numerically higher in CSDH patients compared to control individuals [28].

As previously mentioned, coagulation is linked to the complement system and cross-talk between these systems can lead to both activation and inhibition [15]. For example, activation of the complement system leads to increased pro-coagulant state of platelets [29]. However, many experiments linking coagulation to complement have been performed in vitro many decades ago, leaving the in vivo situation less well explored until more recently [30]. Later, genetically modified rodent models were used to explore the interplay further. For instance, the role of thrombin in relation to C5 cleavage was tested, where C5a was generated even though the mice were C3 deficient and C5a formation was blocked by thrombin inhibitors [31]. C3 seems to play a role in platelet function, since impaired platelet function was observed in C3 deficient mice [32]. Theoretically, in our study, complement factors could have been reduced in CSDH fluid due to depletion depending on an interplay with the coagulation system, but low levels of C5a in the CSDH fluid as well as the dura and membrane rather indicate lack of complement activation at the time point of surgery.

Our study has the strength that the CSDH fluid and the peripheral blood samples were collected at the same time and that the patients were not under general anesthesia prior to retrieval of samples. All samples were analyzed at the same occasion, reducing the risk of bias due to technical differences. The patient cohort is rather small, and recruiting more patients, including those patients with recurrences, will be necessary in future studies. In continued studies, it would also be interesting to compare complement factors in the peripheral circulation from patients with CSDH to healthy controls.

Emerging data have highlighted that CSDH is indeed more than a collection of blood, and that the pathophysiology behind CSDH formation is complex. One hypothesis is that initially, injury mechanisms are activated that may contribute to CSDH progression. Over time, regulatory mechanisms come in place. One such possible regulation seems to be complement inhibition, which might potentially reduce effective clearance of the products in the CSDH. The complement system has a vital role in bridging adaptive and innate immune response, and is connected to the coagulation system. Our data with two factors increased in CSDH fluid argue for an increased production in the capsule and/or adjacent dura, or from recurrent microhemorrhages released into the hematoma. In contrast, the factors decreased in hematoma fluid could reflect degradation in the hematoma or dilution by osmotic and other factors. An inherent limitation of studies on CSDH fluid, is that the samples are obtained at one time, and a highly variable time post-onset of the hematoma formation, which might could reflect different maturation of the CSDH and differences in the inflammatory response [33]. We cannot exclude that the concentration of the analyzed factors in our present study could be different at other time points post-onset of CSDH formation. It would have been valuable to add positive controls to the immunohistochemistry, but achieving tissue for this was not within the ethical permission for this study. Including negative controls was informative though, since there was no staining in negative controls, whereas weak detection of C5a was present in samples with both primary and secondary antibody. Our present work could possibly lay the ground for further work regarding complement in CSDH with the aim to understand whether there are factors that could be inhibited or stimulated in order to improve the clearance of the CSDH.

## Conclusions

Taken together, our data indicate that complement system is inhibited in CSDH fluid upon the time point of surgery. Clusterin, which is an effective inhibitor of complement activation, was increased in the CSDH fluid compared to peripheral blood, whereas factor C9 and factor C5a were decreased. Immunohistochemistry demonstrated no C5b-C9 staining in the CSDH capsule. One possibility is that complement inhibition leads to impaired clearance of debris and a secondary pro-inflammatory response in CSDH.

## Data Availability

No datasets were generated or analysed during the current study.

## List of Abbreviations

A2M: Alpha-2-Macroglobulin
ApoA1: Apolipoprotein A1
ApoC3: Apolipoprotein C3
ASDH: Acute Subdural Hematoma
BBB: Blood-Brain Barrier
Ca1: Carbonic Anhydrase 1
CRP: C-Reactive Protein
CSDH: Chronic Subdural Hematoma
CSF: Cerebrospinal Fluid
DPPIV: Dipeptidyl Peptidase-4
ICAM-1: Intercellular Adhesion Molecule 1
MAC: Membrane Attack Complex
NGAL/LCN2: Neutrophil gelatinase-associated lipocalin /Lipocalin 2
RBP4: Retinol binding protein 4
SAA: Serum amyloid A
SAH: Subarachnoid Hemorrhage
SHBG: Sex hormone binding globulin
sTfR-1: Soluble transferrin receptor-1
tPA: Tissue Plasminogen Activator
VCAM-1: Vascular cell adhesion protein-1
VEGF: Vascular Endothelial Growth Factor
vWf: Von Willebrand factor
